# Efficacy and safety of nalbuphine vs. pethidine in oocyte retrieval: a non-inferiority, double-blinded, randomized controlled trial

**DOI:** 10.3389/frph.2026.1790062

**Published:** 2026-04-30

**Authors:** Pitchapa Em-eam, Marvin Thepsoparn, Chanakarn Suebthawinkul

**Affiliations:** 1 Department of Obstetrics and Gynecology, Faculty of Medicine, Chulalongkorn University, Bangkok, Thailand; 2 King Chulalongkorn Memorial Hospital, Thai Red Cross Society, Bangkok, Thailand; 3 Division of Pain Management, Department of Anesthesiology, Faculty of Medicine and King Chulalongkorn Memorial Hospital, Thai Red Cross Society, Bangkok, Thailand; 4 Division of Reproductive Medicine, Department of Obstetrics and Gynecology, Faculty of Medicine, Chulalongkorn University, Bangkok, Thailand

**Keywords:** nalbuphine, oocyte retrieval, operator score, pain score, pethidine

## Abstract

**Objective:**

The purpose of this study was to evaluate the efficacy and safety of Nalbuphine compared to Pethidine for pain relief during oocyte retrieval.

**Methods:**

In a double-blinded, non-inferiority randomized controlled trial (*n* = 94), participants were randomized equally into two groups. The intervention group received 10 mg intravenous Nalbuphine combined with 5 mg of Midazolam, while the control group received 50 mg intravenous Pethidine with 5 mg of Midazolam. The primary outcome was pain score measured immediately postoperatively using a visual analogue scale. Secondary outcomes included pain score at 2 and 6 h postoperatively, operator score for patient cooperation, vital signs, number of total oocytes and metaphase II (MII) oocytes retrieved, operative time, recovery time, side effects, and need for rescue analgesia.

**Results:**

Nalbuphine demonstrated non-inferiority to Pethidine for pain control at 0 h postoperatively, with a mean difference of 0.52 (95% CI: −0.40 to 1.44). Similar findings were observed at 2 h (mean difference −0.46; 95% CI: −1.30 to 0.38), while at 6 h Nalbuphine showed significantly lower pain scores (mean difference −1.28; 95% CI: −2.15 to −0.41). No significant differences were observed between groups in operator score, recovery time, or rescue analgesia requirements. The number and quality of oocytes retrieved were not affected by the analgesic regimen. Side effects such as nausea, vomiting, dizziness, and tachycardia were compared and showed no significant differences.

**Conclusion:**

Nalbuphine demonstrated non-inferior analgesia to Pethidine for oocyte retrieval with a favorable safety profile. Its impact on long-term reproductive outcomes and clinical superiority warrants further investigation.

**Clinical Trial Registration:**

https://www.thaiclinicaltrials.org/show/TCTR20240406003, identifier TCTR20240406003.

## Introduction

Assisted Reproductive Technology (ART) is a key approach in managing infertility. Conditions including tubal factor infertility, male factor infertility, reduced ovarian reserve, ovarian failure (utilizing donor eggs), and unexplained infertility are predominantly addressed through *in vitro* fertilization (IVF) or intracytoplasmic sperm injection (ICSI) (1).

Oocyte retrieval is an essential technique in assisted reproductive technology (ART) for infertility and facilitates fertility preservation for individuals wishing to retain eggs for future use, including patients undergoing cancer treatment and even LGBTQ + individuals (2, 3). Using transvaginal ultrasonography (TVS)-guided, a needle was inserted through the vaginal wall to retrieve the egg. Even though this is a minor operation, usually lasting 15–30 min and seldom requiring general anesthesia, the procedure may still produce unpleasant pain for patients. Therefore, analgesia is usually used throughout the procedure (4–6).

Pethidine 50 mg combined with Midazolam 5 mg administered intravenously is a commonly used regimen for oocyte retrieval at King Chulalongkorn Memorial Hospital, Bangkok, Thailand. This is administered 10 min prior to the procedure to provide analgesia and sedation during the operation.

Pethidine, a mu (*μ*)-opioid receptor agonist, is commonly used to manage moderate to severe pain. Nevertheless, its use is limited by some undesirable side effects such as nausea and vomiting, respiratory depression, bradycardia, hypotension, and the potential for addiction (7–9). However, Pethidine is widely used for brief gynecologic and minor outpatient procedures in Thailand due to its rapid onset, short duration of action, and familiarity among clinicians.

Nalbuphine, on the other hand, is a synthetic agonist-antagonist opioid approved by the United States and the Thai Food and Drug Administration for the treatment of moderate to severe pain. It acts as an antagonist at mu (*μ*)-opioid receptors and an agonist at kappa (*κ*) -opioid receptors. Nalbuphine, in contrast to Pethidine, is not categorized as a narcotic or prohibited substance in Thailand and possesses a diminished risk of addiction. A notable safety advantage is its ceiling effect on respiratory depression; beyond a certain dosage, additional increments do not substantially exacerbate respiratory depression. Compared with other opioids, Nalbuphine produces significantly less respiratory suppression, making it particularly advantageous in outpatient settings where rapid recovery and patient safety are priorities. Frequently reported adverse effects include drowsiness, dry mouth, and sweating (10–15).

While various conscious sedation and analgesic regimens have been studied for alleviating pain during oocyte retrieval, existing evidence does not endorse any specific method or technique as superior in delivering effective conscious sedation and analgesia for pain relief during and post-procedure. Furthermore, no prior studies have directly compared Nalbuphine with Pethidine in conjunction with Midazolam (16–19). Therefore, this study aims to evaluate the efficacy of Nalbuphine as a substitute for Pethidine in providing analgesia during oocyte retrieval procedures.

## Materials and methods

### Study design

This is a double-blinded, non-inferiority randomized controlled trial conducted at the infertility clinic of King Chulalongkorn Memorial Hospital, Bangkok, Thailand. The study period was from May 2024 to May 2025, including a pilot study during May–July 2024. The study protocol was approved by ﻿the Institutional Review Board, Faculty of Medicine, Chulalongkorn University, Bangkok, Thailand (IRB No. 0873/66), and was registered with the Thai Clinical Trials Registry on 6 April 2024 (TCTR20240406003). The CONSORT guidelines 2025 were followed in this study. All participants provided written informed consent prior to oocyte retrieval. All methods were performed in accordance with the relevant guidelines and regulations.

The study population included women aged 18 to 48 years undergoing oocyte retrieval for IVF/ICSI, fertility preservation, oocyte donation, or other medical indications. The exclusion criteria included active liver disease, uncontrolled asthma, known allergy to Nalbuphine, Pethidine, or Midazolam, recent monoamine oxidase inhibitor usage within two weeks, and fewer than three ovarian follicles seen using TVS on the day of ovulation triggering.

### Ovarian stimulation protocol and intervention

﻿Ovarian stimulation was performed as previously described (20–23). The patients were prescribed either recombinant follicle-stimulating hormone (rFSH) or human menopausal gonadotropin (hMG) for ovarian stimulation. The flexible GnRH antagonist protocol was implemented for pituitary suppression. After individualized ovarian stimulation, either recombinant or urinary human chorionic gonadotropin or ﻿0.2 mg GnRH agonist was administered when at least three follicles reached a mean diameter of 18 mm on TVS. Oocytes were retrieved 36–37 h after ovulation triggering.

All participants were randomized in a 1:1 ratio using blocks of four into either the Nalbuphine group or the Pethidine group. The medications were prepared by a nurse who was not involved in data collection or outcome evaluation. In the Pethidine group, 50 mg of Pethidine was combined with 5 mg of Midazolam and diluted with normal saline to achieve a total volume of 10 mL. In the Nalbuphine group, 10 mg of Nalbuphine was combined with 5 mg of Midazolam and subsequently diluted to a volume of 10 mL. Both preparations were colorless, and each syringe was sealed in an envelope with the participant's ID.

Intravenous access was established before drug administration. The assigned regimen was administered intravenously by medical staff uninvolved in data collection or outcome evaluation 10 min before the oocyte retrieval procedure. Both the participants and the operating physicians were blinded to the treatment allocation.

Throughout the procedure and during the immediate postoperative period, vital signs including blood pressure, heart rate, respiratory rate, and oxygen saturation were continuously monitored and recorded at predefined time points. If a participant reported intolerable pain during the procedure, a rescue dose (half of the originally assigned drug) was administered intravenously and was recorded.

After the procedure, all patients were monitored in the recovery room. They were discharged upon attaining a Modified Post-Anesthesia Discharge Scoring System (MPADSS) score of 9 or above, with the recovery period documented. After discharge, patients were allowed to take Paracetamol at a dosage of 10–15 mg/kg every 4–6 h as needed and were asked to report the total amount during follow-up. Postoperative assessments were conducted via telephone at 2 and 6 h to evaluate pain scores using the visual analogue scale (VAS), and again at 24 h to assess for any adverse effects or complications.

### Outcome measures

The primary outcome was immediate postoperative pain (0 h), assessed using a VAS (0–10). Secondary outcomes included pain score at 2 and 6 h postoperatively, operator-rated patient cooperation (0–10 scale; 0 = worst, 10 = best), vital signs (measured at 10 min post-injection, and at 0, 30, and 60 min post-procedure), number of oocytes retrieved and number of Metaphase II oocytes, recovery time, the need for rescue analgesia both intraoperatively and postoperatively, adverse events, and the dosage of paracetamol taken until 24 h postoperatively.

### Sample size calculation

Sample size was calculated using data from the pilot study (*N* = 16). With a mean pain score of 2.75 (SD = 2.81) in the Pethidine group and 2.81 in the Nalbuphine group (SD = 2.71), a non-inferiority margin of 1.5, an alpha of 0.05, and 80% power, a total sample size of 94 participants (47 per group) was determined to be sufficient.

### Statistical analyses

Statistical analyses were performed using SPSS version 29.0.1.0 (IBM Corp., Armonk, NY, USA). Continuous variables were expressed as mean ± standard deviation (SD) for normally distributed data or median and interquartile range (IQR) for non-normally distributed data. Categorical variables were reported as frequency and percentage. The primary outcome was analyzed using a non-inferiority test with a predefined margin of 1.5. Between-group comparisons of continuous variables were performed using the independent t-test or Mann–Whitney U test, as appropriate. Categorical data were compared using the Chi-square test or Fisher's exact test. Vital signs across time points within treatment group were analyzed using two-way repeated measures ANOVA via the General Linear Model followed by Bonferroni-adjusted multiple comparsion tests. Vital signs across the groups were analyzed without formal adjustment for multiple comparisons. A *p*-value < 0.05 was considered statistically significant. All randomized participants completed the study and were included in the final analysis, consistent with both intention-to-treat and per-protocol principles.

## Results

**Figure 1:**
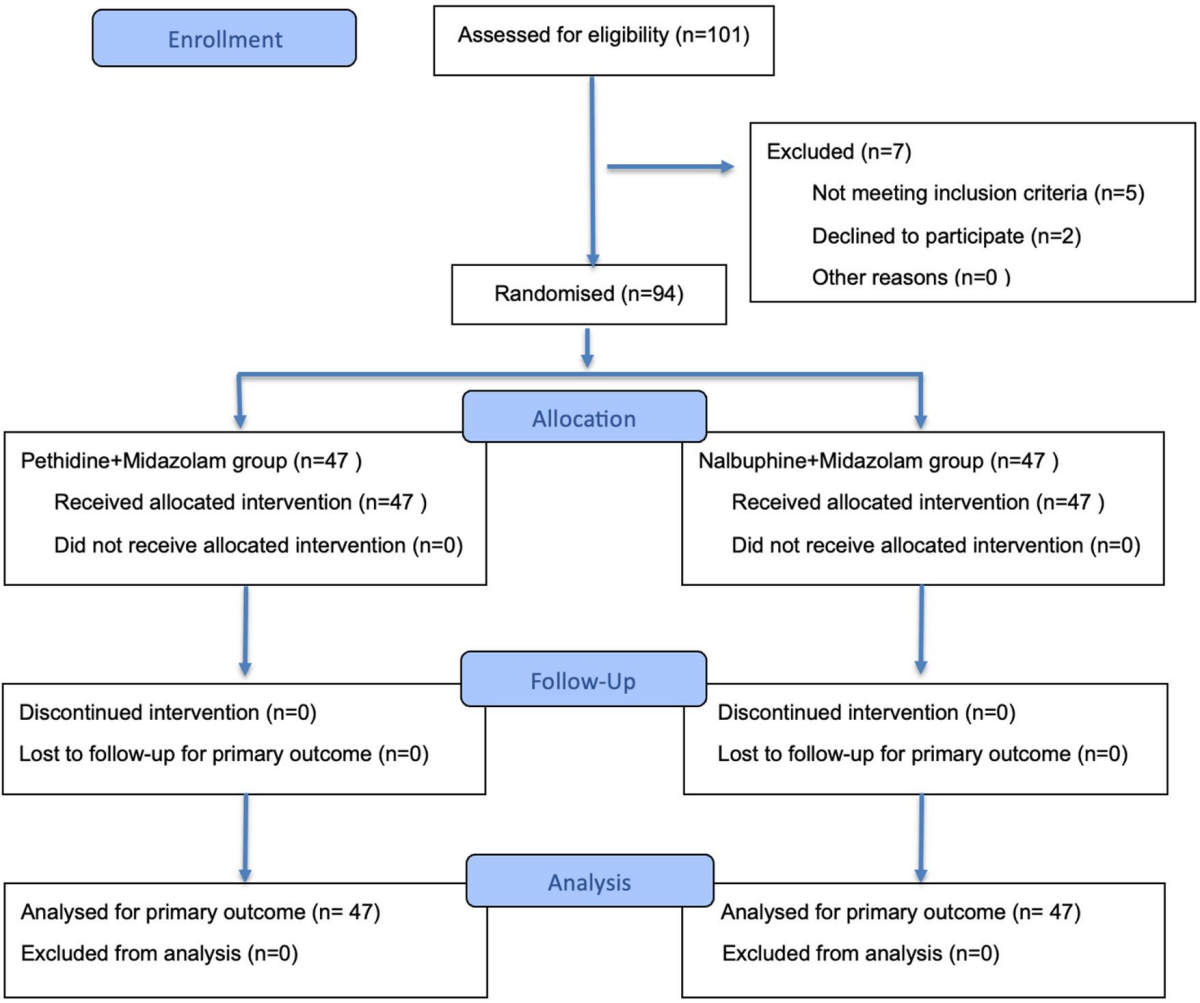
CONSORT 2025 flow diagram. Flow diagram of the progress through the phases of a randomized trial of two groups (that is, enrolment, intervention allocation, follow-up, and data analysis).

A total of 101 participants were screened for eligibility. The remaining 94 participants were randomized equally into the Pethidine group (n = 47) and the Nalbuphine group (*n* = 47). None of the patients was lost to follow-up or failed to follow the protocol, and all participants were included in the final analysis (Figure 1). Both intention-to-treat (ITT) and per-protocol (PP) analyses were prespecified. The ITT population included all randomized participants analyzed according to assigned treatment. The PP population included participants who completed the study without major protocol deviations. As no protocol deviations, crossovers, or losses to follow-up occurred, the ITT and PP populations were identical.

Baseline demographic and clinical characteristics of patients are displayed in Table 1. There was no difference between the two groups in age, body mass index, baseline hormone levels including Estradiol (E2), Luteinizing hormone (LH), Follicle-stimulating hormone (FSH), Prolactin (PRL), Anti-Mullerian hormone (AMH), underlying disease, previous pregnancy, prior abdominal surgery, and causes of infertility.

**Table 1:** Baseline characteristics (*n* = 47 per group).

Baseline Characteristic	Pethidine (*n* = 47)	Nalbuphine (*n* = 47)
	Mean ± SD	Mean ± SD
Age (year)	37.34 ± 3.99	37.15 ± 4.13
Body weight (kg)	58.38 ± 12.24	60.16 ± 11.81
Height (cm)	158.69 ± 5.19	159.83 ± 5.31
Body Mass Index (kg/m^2^)	23.21 ± 4.92	23.47 ± 3.88
Class (n,%)		
- Underweight (<18.5)	5 (10.6%)	4 (8.5%)
- Normal (18.5–22.99)	25 (53.2%)	18 (38.3%)
- Overweight (23–24.99)	3 (6.4%)	12 (25.5%)
- Obesity class I (25–29.99)	7 (14.9%)	10 (21.3%)
- Obesity class II (≥ 30)	7 (14.9%)	3 (6.4%)
Baseline hormones		
Estradiol (pg/mL)	45.13 ± 20.16	57.24 ± 57.61
LH (IU/L)	3.78 ± 2.04	3.66 ± 1.96
FSH (IU/L)	6.47 ± 3.19	6.66 ± 2.28
PRL (ng/mL)	12.31 ± 5.0	12.88 ± 6.38
AMH (ng/mL)	2.17 ± 2.23	2.14 ± 1.38
Underlying disease (n,%)		
- None	38 (80.9%)	36 (76.6%)
- Thyroid disease	4 (8.5%)	3 (6.4%)
- Hepatitis B virus carrier	2 (4.3%)	3 (6.4%)
- Dyslipidemia	3 (6.4%)	0 (0%)
- Polycystic Ovarian Syndrome	2 (4.3%)	1 (2.1%)
- Migraine	1 (2.1%)	1 (2.1%)
- Hypertension	1 (2.1%)	0 (0%)
- Diabetes Milletus	1 (2.1%)	0 (0%)
- Cancer	1 (2.1%)	0 (0%)
- Major depressive disorder	0 (0%)	1 (2.1%)
- Allergic Rhinitis	0 (0%)	1 (2.1%)
- Disc herniation	0 (0%)	1 (2.1%)
- Arthritis	0 (0%)	1 (2.1%)
Previous Pregnancy (*n*,%)		
- No	34 (72.3%)	33 (70.2%)
- Yes	13 (27.7%)	14 (29.8%)
Previous abdominal surgery (*n*,%)		
- No	33 (70.2%)	37 (78.7%)
- Yes	14 (29.8%)	10 (21.3%)
Cause of infertility (*n*,%)		
- Mix/Unknown/Other	22 (46.8%)	29 (61.7%)
- Male	14 (29.8%)	4 (8.5%)
- Endometriosis	4 (8.5%)	6 (12.8%)
- Donor/Social freezing	3 (6.4%)	5 (10.6%)
- Tubal	4 (8.5%)	3 (6.4%)

LH, Luteinizing hormone; FSH, Follicle stimulating hormone; PRL, Prolactin; AMH, Anti-Mullerian hormone.

BMI was classified by World Health Organization Asian-specific BMI (aBMI) classification.

### Primary outcome: pain score

**Figure 2:**
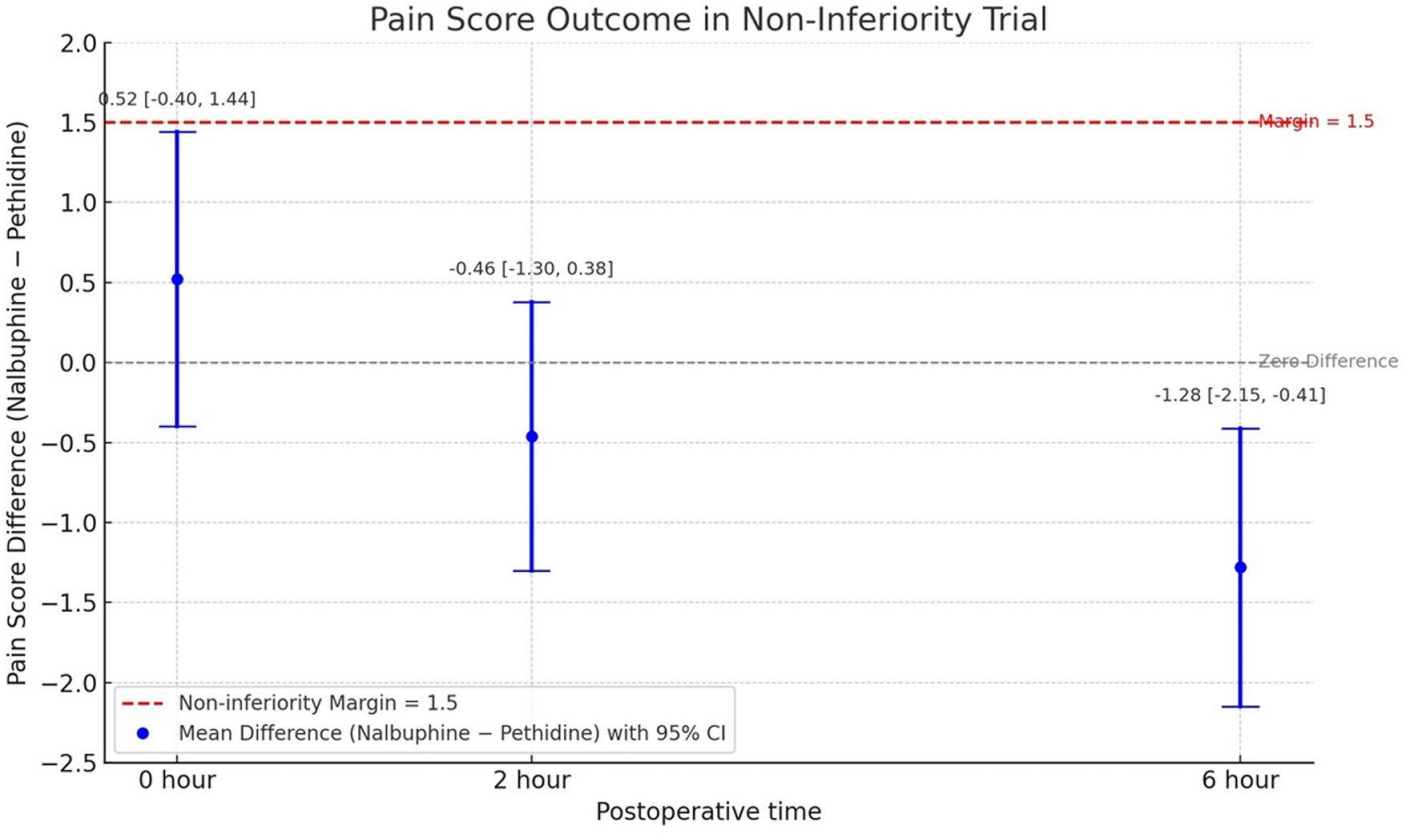
Pain score outcome at 0, 2, and 6 h postoperative between nalbuphine group and pethidine group. Mean differences and 95% confidence intervals of postoperative pain scores at 0, 2, and 6 h after oocyte retrieval between the Nalbuphine and Pethidine groups. Pain was assessed using a visual analogue scale (0 = no pain, 10 = worst possible pain).

Pain scores were assessed using the VAS (0–10). The test of non-inferiority demonstrated that pain scores in the Nalbuphine group were non-inferior to those in the Pethidine group at all time points. At 0 h post-procedure, the mean difference was 0.52 ± 0.92 (95% CI: −0.40 to 1.44). At 2 h, the mean difference was −0.46 ± 0.84 (95% CI: −1.3 to 0.38). At 6 h, the mean difference was −1.28 ± 0.87 (95% CI: −2.15 to −0.41), favoring Nalbuphine (Figure 2).

### Secondary outcomes

#### Vital signs

**Figure 3:**
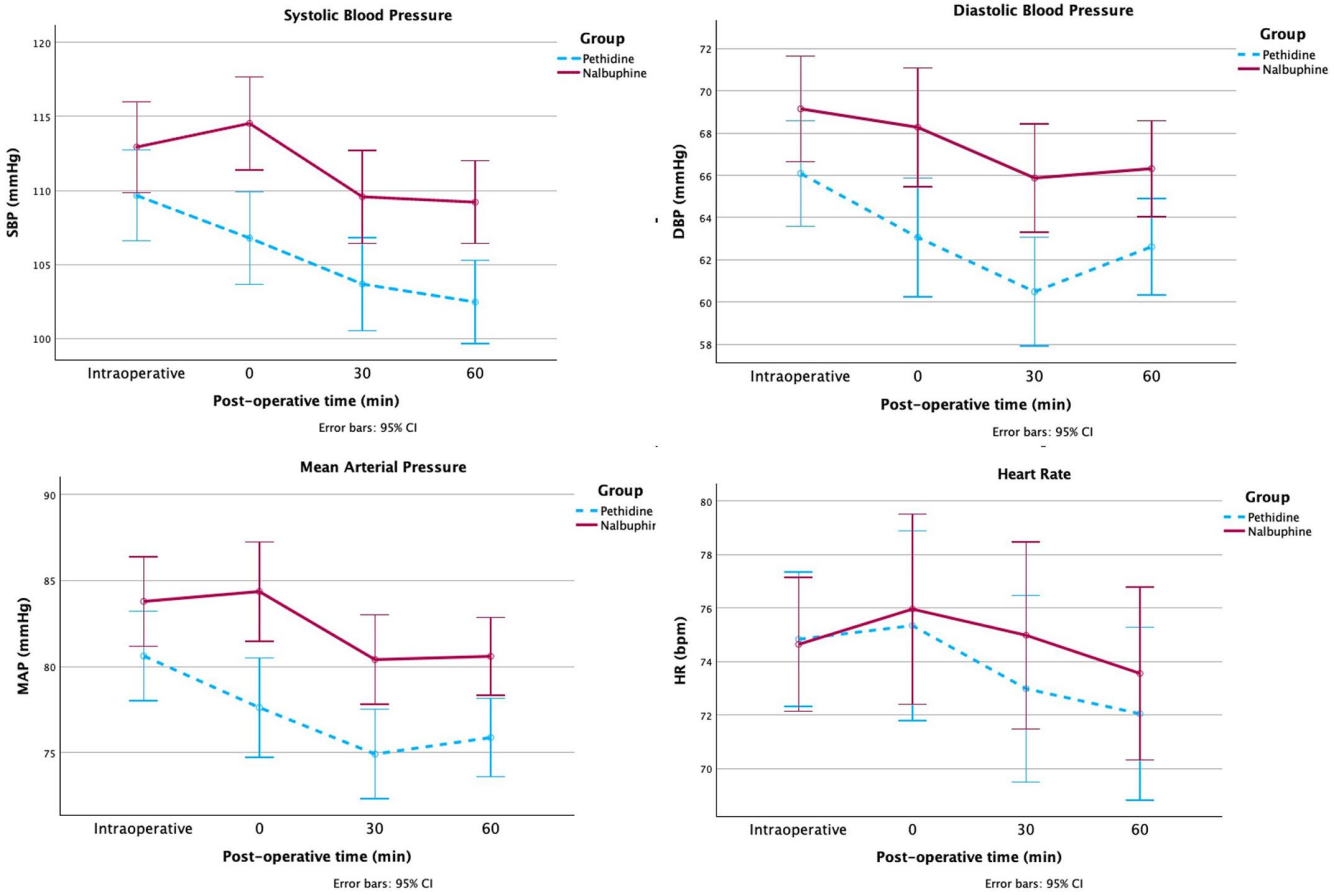
Blood pressure and heart rate at intraoperative and postoperative 0, 30, and 60 min between nalbuphine group and pethidine group *intraoperative = 10 min after administration of medication.

Vital signs were recorded at the start of the procedure and at 0, 30, and 60 min postoperatively. Two-way repeated-measures ANOVA demonstrated a significant effect of time on systolic, diastolic, and mean arterial pressures (all *p* < 0.001), indicating expected hemodynamic changes over the perioperative period. However, there was no significant group-by-time interaction for any blood pressure parameter, suggesting comparable hemodynamic trends between the Nalbuphine and Pethidine groups (Figure 3).

Although nominal differences in systolic, diastolic, and mean arterial pressures were observed at certain postoperative time points (Supplementary Table 1), these should be interpreted cautiously, as the overall interaction effects were not statistically significant. Within-group pairwise comparisons (Bonferroni-adjusted) showed a reduction in diastolic blood pressure in the Pethidine group between the start of the procedure and 30 min postoperatively [mean difference = 5.60 mmHg, 95% CI (1.95, 9.24), *p* < 0.001], as well as a modest decrease in heart rate between 0 and 60 min [mean difference = 3.30 bpm, 95% CI (0.08, 6.52), *p* = 0.042]. However, given the absence of a significant group-by-time interaction, these within-group changes should be interpreted as time-related effects rather than evidence of differential treatment effects.

The respiratory rate and oxygen saturation remained stable between groups and across time points. All recorded hemodynamic values remained within clinically acceptable ranges, and no patient required medical intervention.

#### Operator-rated patient cooperation

Operator-rated patient cooperation score, assessed using a 0–10 scale (0 = worst, 10 = best), showed no significant difference between groups [media*n* = 8 (IQR 7–9) in both groups; *p* = 0.601] (Table 2).

**Table 2:** Operative outcomes between nalbuphine group and pethidine group.

Outcomes	Pethidine (*n* = 47)	Nalbuphine (*n* = 47)	*P*-value*
	Median (Q1; Q3)	Median (Q1; Q3)	
Operator score	8 (7;9)	8 (7;9)	0.601
Number of oocytes retrieved (n)	11 (6;14)	11 (5;15)	0.820
Metaphase II (MII) oocyte (n)	8 (4;12)	8 (4;12)	0.862
Outcomes	Mean ± SD	Mean ± SD	*P*-value**
%MII	74.46 + 18.23	70.21 + 23.94	0.336
Operative time (min)	13.15 ± 4.27	12.09 ± 3.55	0.193
Recovery time (min)	76.91 ± 9.81	75.74 ± 7.87	0.525
Side Effects (*n*, %) ***			
- None	12 (74.5%)	17 (63.8%)	0.264
- Somnolence	7 (14.9%)	4 (8.5%)	0.523
- Nausea/Vomiting	13 (27.7%)	22 (46.8%)	0.055
- Dizziness	15 (31.9%)	16 (34.0%)	0.826
- Tachycardia	2 (4.3%)	1 (2.1%)	1.000
- Hypotension	7 (14.9%)	2 (4.3%)	0.158
- Acute urinary retention	1 (2.1%)	1 (2.1%)	1.000
- Dry mouth/throat	1 (2.1%)	0 (0%)	1.000
- Bradycardia	7 (14.9%)	4 (8.5%)	0.523
- Chest discomfort	1 (2.1%)	0 (0%)	1.000
Rescue drug (*n*, %)***			
- None	31 (66%)	30 (63.8%)	0.829
- Intraoperative	2 (4.3%)	3 (6.4%)	0.646
- Postoperative	15 (31.9%)	16 (34%)	0.826
○ Paracetamol dose (mg) [Median (Q1; Q3)]	500 (500; 2,000)	750 (500; 1,875)	0.761

*Mann–Whitney U test **Unpaired T-test***Chi-square test or Fisher's exact test as appropriate.

#### Procedural outcomes

No significant differences were observed between groups regarding the number of oocytes retrieved or the number and percentage of MII oocytes, operative time, or recovery time. The median number of oocytes retrieved was 11 in both groups (*p* = 0.820), and the mean percentage of MII oocytes was 74.5 ± 18.2% in the Pethidine group vs. 70.2 ± 23.9% in the Nalbuphine group (*p* = 0.336). Mean recovery time did not show a statistically significant difference between the groups (76.9 ± 9.8 vs. 75.7 ± 7.9 min; *p* = 0.525) (Table 2).

#### Adverse effects and rescue medication

The incidence of adverse effects was not significantly different between the groups. Common symptoms included nausea/vomiting, dizziness, and somnolence. Intraoperatively, rescue medication was administered to 6.4% of patients in the Nalbuphine group and 4.3% in the Pethidine group, while postoperative rescue analgesia was required in 34.0% and 31.9%, respectively; both comparisons showed no statistical significance. The median postoperative paracetamol dosage was 750 mg (range: 500–4,000 mg) in the Nalbuphine group and 500 mg (range: 500–3,000 mg) in the Pethidine group (*p* = 0.761) (Table 2).

## Discussion

This randomized controlled trial demonstrates that Nalbuphine is non-inferior to Pethidine in providing analgesia during oocyte retrieval, with no significant difference in patient cooperation, side effect profiles, and procedural outcomes. Nalbuphine also demonstrated a more elevated blood pressure profile across multiple postoperative time points.

In our study, pain scores at 0 and 2 h post-procedure did not significantly differ between groups and remained within the predefined non-inferiority margin. Although a statistically significant difference was observed at 6 h postoperatively, this was a secondary outcome and should be interpreted cautiously. Pain at 6 h was assessed by telephone following discharge, which may introduce recall bias and variability in patient circumstances. Additionally, postoperative analgesic use after discharge was not strictly standardized, potentially confounding late pain assessment. While the longer half-life of nalbuphine (3.2 h vs. 5 h for Pethidine and Nalbuphine, respectively) (8, 24) may offer a pharmacologic explanation for this finding, attribution of the 6-hour difference solely to the study drug cannot be confirmed. Therefore, this observation should be considered exploratory rather than evidence of superior analgesic duration. Overall, these findings indicate that Nalbuphine was not inferior to Pethidine for immediate postoperative pain control within the prespecified margin.

In analgesic efficacy trials utilizing a 0–10 pain scale, a 1.5-point non-inferiority margin is methodologically and clinically defensible, aligning with established precedents and the specific nature of pain perception (25–27). This threshold is consistent with the Minimal Clinically Important Difference (MCID) for acute pain—typically cited as 1–2 points—ensuring that the margin remains relevant to the patient experience, particularly when the active control demonstrates a robust effect over placebo. Ultimately, selecting this 1.5-point margin emphasizes clinical equivalence by acknowledging that minor numerical variances do not necessarily represent a perceptible loss in efficacy, thereby allowing for a more holistic evaluation of a treatment's value, including its safety and tolerability profiles.

The reduced systolic, diastolic, and mean arterial pressures observed in the Pethidine group may be attributed to the pharmacodynamic properties of each medication. Pethidine, a pure mu (*μ*)-opioid receptor agonist, is recognized for inducing peripheral vasodilation through histamine release and central sympatholytic effect, potentially leading to hypotension (7, 28–30). On the other hand, Nalbuphine, a combined agonist-antagonist [kappa (*κ*)-agonist and mu (*μ*)-antagonist], exhibits less vasodilatory action and ﻿ appears to preserve sympathetic tone, which may be associated with smaller changes in blood pressure. However, higher blood pressure values are not inherently advantageous, and no predefined criteria for hemodynamic superiority were established in this study. Therefore, these findings should be interpreted as pharmacodynamic differences rather than evidence of clinical benefit.

The Pethidine group also demonstrated a greater reduction in heart rate compared with those in the Nalbuphine group. This finding may be multifactorial. Initially, oocyte retrieval may induce visceral pain that stimulates vasovagal reaction. This response induces transient parasympathetic activation and inhibition of sympathetic tone, producing bradycardia and hypotension (31). In this context, an analgesic that more effectively controls pain may attenuate the reaction. Nalbuphine, being a kappa (*κ*)-receptor–mediated analgesic, may have provided more effective suppression of the visceral pain (32, 33), thereby diminishing vagal activation and exerting a lesser impact on heart rate. Second, Pethidine's action as a mu (*μ*)-opioid receptor agonist may contribute to heart rate reduction via mu (*μ*)-receptor activation in the medullary cardiovascular centers, which might diminish sympathetic outflow, resulting in a central sympatholytic effect (21–23). In outpatient settings where cardiovascular stability is desirable, Nalbuphine's hemodynamic characteristics may provide a clinical advantage.

Our findings align with previous study by Brock-Utne et al. (34), who evaluated intramuscular Nalbuphine 20 mg against Pethidine 100 mg in postoperative orthopedic patients. The study revealed comparable pain scores among groups initially, but demonstrated enhanced analgesia with Nalbuphine after 180 and 360 min following injection. Similarly, Hew et al. (35) compared Nalbuphine 20 mg IV and 40 mg IV with Pethidine 75 mg IM in 150 postoperative patients and found equivalent analgesic efficacy across regimens, with peak effect occurring earlier for Nalbuphine (15 min vs. 30 min with Pethidine). Notably, the incidence of nausea and vomiting in that study was significantly lower with Nalbuphine 20 mg (2%) than with Pethidine 75 mg (22%, *p* < 0.01), indicating a more favorable side-effect profile. These parallel findings strengthen the external validity of our study. In contrast, our study found no significant differences between groups in the incidence of adverse events such as somnolence, nausea, and dizziness. Although Nalbuphine has an established ceiling effect on respiratory depression, our investigation found no significant changes in respiratory rate or oxygen desaturation across the groups. This may result from the relatively low incidence of such events in this setting, and our sample size may not have been adequately powered to detect infrequent respiratory complications.

The requirements for rescue analgesia and postoperative paracetamol consumption were similar between groups, supporting the finding that Nalbuphine did not result in clinically unacceptable reductions in analgesic efficacy compared with Pethidine. Importantly, the analgesic regimen did not influence oocyte yield, maturation rates, or surgical and recovery times, hence reinforcing the procedural safety of Nalbuphine. Consistent with our findings, Liu et al. (19) evaluated 400 women undergoing ultrasound-guided oocyte retrieval and demonstrated that intravenous Nalbuphine (0.1 mg/kg), administered with propofol and remifentanil, provided stable anesthesia and effective analgesia without compromising embryo quality or pregnancy outcomes. Together, these findings support Nalbuphine as an effective and safe alternative to Pethidine for oocyte retrieval, although larger multicenter trials with long-term follow-up might be needed to confirm its reproductive safety.

This study has several strengths, including being the first double blinded, randomized controlled trial comparing Nalbuphine with Pethidine during oocyte retrieval, a sufficient sample size derived from a pilot study, with no drop out rate, and a thorough evaluation of both clinical efficacy and safety. The limitation is the reliance on patient-reported pain scores obtained via telephone at 2 and 6 h postoperatively, which may introduce recall bias. Other limitations of this study include the lack of assessment of the cost-effectiveness of Nalbuphine compared to Pethidine in treatment cycles, as well as the absence of investigation into the long-term safety of Nalbuphine concerning embryo quality and pregnancy outcomes, which we assumed could be influenced by sperm quality and individual variables. Although the number of retrieved oocytes and proportion of MII oocytes did not differ between groups, downstream reproductive outcomes such as fertilization rate, embryo development, implantation, and pregnancy outcomes were not evaluated in this study. However, prior research, notably Liu et al. (19), have not demonstrated detrimental effects of Nalbuphine on ART outcomes. Future research with longer follow-up could be needed to confirm reproductive safety.

In summary, Nalbuphine is a safe and non-inferior to Pethidine for analgesia during oocyte retrieval. Its advantageous side effect profile and consistent hemodynamic response render it an appropriate option, particularly in outpatient or resource-limited settings. Further studies may validate these findings and encourage long-term safety as well as wider clinical implementation of Nalbuphine for the oocyte retrieval procedure.

## Conclusions

Nalbuphine demonstrated non-inferior analgesia to Pethidine for oocyte retrieval, with a favorable safety profile and stable hemodynamic response. While these findings support Nalbuphine as a viable clinical alternative, further studies are warranted to evaluate its impact on long-term reproductive outcomes and to establish its potential clinical superiority.

## Attestation statement

- The subjects in this trial have not concomitantly been involved in other randomized Trials.
- Data regarding any of the subjects in the study has not been previously published unless specified.
- Data will be made available to the editors of the journal for review or query upon request.

## Data Availability

The raw data supporting the conclusions of this article will be made available by the authors, without undue reservation.
