# Supplementary material for: Efficacy and safety of nalbuphine vs. pethidine in oocyte retrieval: a non-inferiority, double-blinded, randomized controlled trial
Source: Front Reprod Health. 2026 Apr 30;8:1790062. doi: 10.3389/frph.2026.1790062 (PMC13171855; doi:10.3389/frph.2026.1790062)
Supplement: Supplementary file 1 [file Table1.docx]

**Table 1 Baseline characteristics (n= 47 per group)**

| **Baseline Characteristic** | **Pethidine  (n=47)** | **Nalbuphine  (n=47)** |
| --- | --- | --- |
|  | **Mean + SD** | **Mean + SD** |
| **Age (year)** | 37.34 + 3.99 | 37.15 + 4.13 |
| **Body weight (kg)** | 58.38 + 12.24 | 60.16 + 11.81 |
| **Height (cm)** | 158.69 + 5.19 | 159.83 + 5.31 |
| **Body Mass Index (kg/m^2^)**  **Class (n,%)**   - **Underweight (<18.5)** - **Normal (18.5 – 22.99)** - **Overweight (23 – 24.99)** - **Obesity class I (25 – 29.99)** - **Obesity class II (> 30)** | 23.21 + 4.919  5 (10.6%)  25 (53.2%)  3 (6.4%)  7 (14.9%)  7 (14.9%) | 23.47 + 3.88  4 (8.5%)  18 (38.3%)  12 (25.5%)  10 (21.3%)  3 (6.4%) |
| **Baseline hormones** | | |
| **Estradiol (pg/ml)** | 45.13 + 20.16 | 57.24 + 57.61 |
| **LH (IU/L)** | 3.78 + 2.04 | 3.66 + 1.96 |
| **FSH (IU/L)** | 6.47 + 3.19 | 6.66 + 2.28 |
| **PRL (ng/ml)** | 12.31 + 5.0 | 12.88 + 6.38 |
| **AMH (ng/ml)** | 2.17 + 2.23 | 2.14 + 1.38 |
| **Underlying disease (n,%)**   - **None** - **Thyroid disease** - **Hepatitis B virus carrier** - **Dyslipidemia** - **Polycystic Ovarian Syndrome** - **Migraine** - **Hypertension** - **Diabetes Milletus** - **Cancer** - **Major depressive disorder** - **Allergic Rhinitis** - **Disc herniation** - **Arthritis** | 38 (80.9%)  4 (8.5%)  2 (4.3%)  3 (6.4%)  2 (4.3%)  1 (2.1%)  1 (2.1%)  1 (2.1%)  1 (2.1%)  0 (0%)  0 (0%)  0 (0%)  0 (0%) | 36 (76.6%)  3 (6.4%)  3 (6.4%)  0 (0%)  1 (2.1%)  1 (2.1%)  0 (0%)  0 (0%)  0 (0%)  1 (2.1%)  1 (2.1%)  1 (2.1%)  1 (2.1%) |
| **Previous Pregnancy (n,%)**   - **No** - **Yes** | 34 (72.3%)  13 (27.7%) | 33 (70.2%)  14 (29.8%) |
| **Previous abdominal surgery (n,%)**   - **No** - **Yes** | 33 (70.2%)  14 (29.8%) | 37 (78.7%)  10 (21.3%) |
| **Cause of infertility (n,%)**   - **Mix/Unknown/Other** - **Male** - **Endometriosis** - **Donor/Social freezing** - **Tubal** | 22 (46.8%)  14 (29.8%)  4 (8.5%)  3 (6.4%)  4 (8.5%) | 29 (61.7%)  4 (8.5%)  6 (12.8%)  5 (10.6%)  3 (6.4%) |

LH; Luteinizing Hormone, FSH; Follicle Stimulating Hormone, PRL; Prolactin, AMH; Anti-Mullerian Hormone

BMI was classified by World Health Organization Asian-specific BMI (aBMI) classification

**Table 2. Operative outcomes between Nalbuphine group and Pethidine group**

| **Outcomes** | **Pethidine  (n=47)** | **Nalbuphine  (n=47)** | **P-value*** |
| --- | --- | --- | --- |
|  | **Median (Q1; Q3)** | **Median (Q1; Q3)** |  |
| **Operator score** | 8 (7;9) | 8 (7;9) | 0.601 |
| **Number of oocytes retrieved (n)** | 11 (6;14) | 11 (5;15) | 0.820 |
| **Metaphase II (MII) oocyte (n)** | 8 (4;12) | 8 (4;12) | 0.862 |
|  | **Mean + SD** | **Mean + SD** | **P-value**** |
| **%MII** | 74.46 + 18.23 | 70.21 + 23.94 | 0.336 |
| **Operative time (min)** | 13.15 + 4.27 | 12.09 + 3.55 | 0.193 |
| **Recovery time (min)** | 76.91 + 9.81 | 75.74 + 7.87 | 0.525 |
| **Side Effects (n, %)** ***   - **None** - **Somnolence** - **Nausea/Vomiting** - **Dizziness** - **Tachycardia** - **Hypotension** - **Acute urinary retention** - **Dry mouth/throat** - **Bradycardia** - **Chest discomfort** | 12 (74.5%)  7 (14.9%)  13 (27.7%)  15 (31.9%)  2 (4.3%)  7 (14.9%)  1 (2.1%)  1 (2.1%)  7 (14.9%)  1 (2.1%) | 17 (63.8%)  4 (8.5%)  22 (46.8%)  16 (34.0%)  1 (2.1%)  2 (4.3%)  1 (2.1%)  0 (0%)  4 (8.5%)  0 (0%) | 0.264  0.523  0.055  0.826  1.000  0.158  1.000  1.000  0.523  1.000 |
| **Rescue drug (n, %)*****   - **None** - **Intraoperative** - **Postoperative**   - **Paracetamol dose (mg) [Median (Q1; Q3)]** | 31 (66%)  2 (4.3%)  15 (31.9%)  500 (500; 2000) | 30 (63.8%)  3 (6.4%)  16 (34%)  750 (500; 1875) | 0.829  0.646  0.826  0.761 |

*Mann-Whitney U test **Unpaired T-test***Chi-square test or Fisher’s exact test as appropriate

**Table 1 Baseline characteristics (n= 47 per group)**

| **Baseline Characteristic** | **Pethidine  (n=47)** | **Nalbuphine  (n=47)** |
| --- | --- | --- |
|  | **Mean + SD** | **Mean + SD** |
| **Age (year)** | 37.34 + 3.99 | 37.15 + 4.13 |
| **Body weight (kg)** | 58.38 + 12.24 | 60.16 + 11.81 |
| **Height (cm)** | 158.69 + 5.19 | 159.83 + 5.31 |
| **Body Mass Index (kg/m^2^)**  **Class (n,%)**   - **Underweight (<18.5)** - **Normal (18.5 – 22.99)** - **Overweight (23 – 24.99)** - **Obesity class I (25 – 29.99)** - **Obesity class II (> 30)** | 23.21 + 4.919  5 (10.6%)  25 (53.2%)  3 (6.4%)  7 (14.9%)  7 (14.9%) | 23.47 + 3.88  4 (8.5%)  18 (38.3%)  12 (25.5%)  10 (21.3%)  3 (6.4%) |
| **Baseline hormones** | | |
| **Estradiol (pg/ml)** | 45.13 + 20.16 | 57.24 + 57.61 |
| **LH (IU/L)** | 3.78 + 2.04 | 3.66 + 1.96 |
| **FSH (IU/L)** | 6.47 + 3.19 | 6.66 + 2.28 |
| **PRL (ng/ml)** | 12.31 + 5.0 | 12.88 + 6.38 |
| **AMH (ng/ml)** | 2.17 + 2.23 | 2.14 + 1.38 |
| **Underlying disease (n,%)**   - **None** - **Thyroid disease** - **Hepatitis B virus carrier** - **Dyslipidemia** - **Polycystic Ovarian Syndrome** - **Migraine** - **Hypertension** - **Diabetes Milletus** - **Cancer** - **Major depressive disorder** - **Allergic Rhinitis** - **Disc herniation** - **Arthritis** | 38 (80.9%)  4 (8.5%)  2 (4.3%)  3 (6.4%)  2 (4.3%)  1 (2.1%)  1 (2.1%)  1 (2.1%)  1 (2.1%)  0 (0%)  0 (0%)  0 (0%)  0 (0%) | 36 (76.6%)  3 (6.4%)  3 (6.4%)  0 (0%)  1 (2.1%)  1 (2.1%)  0 (0%)  0 (0%)  0 (0%)  1 (2.1%)  1 (2.1%)  1 (2.1%)  1 (2.1%) |
| **Previous Pregnancy (n,%)**   - **No** - **Yes** | 34 (72.3%)  13 (27.7%) | 33 (70.2%)  14 (29.8%) |
| **Previous abdominal surgery (n,%)**   - **No** - **Yes** | 33 (70.2%)  14 (29.8%) | 37 (78.7%)  10 (21.3%) |
| **Cause of infertility (n,%)**   - **Mix/Unknown/Other** - **Male** - **Endometriosis** - **Donor/Social freezing** - **Tubal** | 22 (46.8%)  14 (29.8%)  4 (8.5%)  3 (6.4%)  4 (8.5%) | 29 (61.7%)  4 (8.5%)  6 (12.8%)  5 (10.6%)  3 (6.4%) |

LH; Luteinizing Hormone, FSH; Follicle Stimulating Hormone, PRL; Prolactin, AMH; Anti-Mullerian Hormone

BMI was classified by World Health Organization Asian-specific BMI (aBMI) classification

**Supplementary** **Table 1 Vital signs between Nalbuphine group and Pethidine group**

| **Outcomes** | **Pethidine  (n=47)** | **Nalbuphine  (n=47)** | **P-value** |
| --- | --- | --- | --- |
|  | **Mean + SD** | **Mean + SD** |  |
| **Systolic Blood pressure (mmHg)** | | | |
| Intraoperative | 109.66 + 9.97 | 112.94 + 11.19 | 0.137 |
| 0 min | 106.79 + 11.15 | 114.53 + 10.56 | <0.001* |
| 30 min | 103.68 + 10.10 | 109.57 + 11.50 | 0.010* |
| 60 min | 102.47 + 9.40 | 109.21 + 9.97 | 0.001* |
| **Diastolic Blood pressure (mmHg)** | | | |
| Intraoperative | 66.09 + 8.27 | 69.15 + 8.93 | 0.088 |
| 0 min | 63.06 + 10.05 | 68.28 + 9.39 | 0.011* |
| 30 min | 60.49 + 8.29 | 65.87 + 9.41 | 0.004* |
| 60 min | 62.62 + 7.52 | 66.32 + 8.22 | 0.025* |
| **Mean Arterial Pressure (mmHg)** | | | |
| Intraoperative | 80.62 + 8.56 | 83.79 + 9.39 | 0.090 |
| 0 min | 77.62 + 10.00 | 84.36 + 9.95 | 0.001* |
| 30 min | 74.91 + 8.43 | 81.13 + 10.35 | 0.004* |
| 60 min | 75.87 + 7.34 | 80.60 + 8.31 | 0.004* |
| **Heart rate (bpm)** | | | |
| Intraoperative | 74.83 + 10.45 | 74.64 + 6.43 | 0.915 |
| 0 min | 75.34 + 13.21 | 75.96 + 11.24 | 0.808 |
| 30 min | 72.98 + 11.93 | 74.98 + 12.22 | 0.424 |
| 60 min | 72.04 + 11.21 | 73.55 + 11.11 | 0.513 |
| **Respiratory rate (/min)** | | | |
|  | **Median (Q1;Q3)** | **Median (Q1;Q3)** |  |
| Intraoperative | 18 (17;20) | 18 (18;20) | 0.607 |
| 0 min | 18 (17;20) | 18 (16;20) | 0.373 |
| 30 min | 18 (16;20) | 18 (16;20) | 0.493 |
| 60 min | 18 (18;20) | 18 (16;20) | 0.506 |
| **Oxygen saturation (%)** | | | |
| Intraoperative | 100 (100;100) | 100 (100;100) | 0.079 |
| 0 min | 100 (98;100) | 100 (98;100) | 0.904 |
| 30 min | 100 (99;100) | 100 (98;100) | 0.181 |
| 60 min | 100 (99;100) | 100 (99;100) | 0.602 |

*Statistical significance at level of 0.05
